# The Satisfaction of Women’s Orgasms: The Relationship Between Women’s Orgasmic Pleasure and Sexual Relationship Satisfaction in Aotearoa/New Zealand

**DOI:** 10.1080/19317611.2025.2464535

**Published:** 2025-02-22

**Authors:** Alexandra K. Janssen, Rita I. Csako, David L. Rowland, Krisztina Hevesi

**Affiliations:** a Auckland University of Technology, Auckland, New Zealand; b School of Environment, Education and Development, The University of Manchester, Manchester, UK; c Valparaiso University, Valparaiso, IN; d Institute of Psychology, Eötvös Loránd University, Budapest, Hungary

**Keywords:** Women, orgasms, relationship, satisfaction, sex

## Abstract

**Objectives:**

Orgasms have become a symbol of successful sex in modern society. It is common knowledge that women’s orgasms can be elusive in heterosexual partnered sex. To ascertain the impact of this disparity and to better understand women’s sexuality for clinical applications, this study explores the relationship between women’s orgasms and their relationship satisfaction in Aotearoa/New Zealand.

**Methods:**

This cross-sectional study utilises data from an online questionnaire. A stepwise logistic regression model building exercise was run on select orgasm variables (consistency, pleasure, and difficulty) and covariates (age, frequency of sex, and self-rated importance of sex) to determine which variables best predicted sexual relationship satisfaction.

**Results:**

Notably, more consistent orgasms and more frequent sex were the strongest predictors of sexual relationship satisfaction. The relationship between orgasmic variables and sexual relationship satisfaction was curvilinear, with satisfaction increasing only up to a certain point.

**Conclusions:**

These findings demonstrate an association between women’s orgasms and their relationship satisfaction. Consistent with research in other Western populations, sex and relationship therapists working with women aiming to improve their sexual relationship satisfaction might achieve better outcomes by focusing on interventions that increase the frequency of sex and/or improve orgasm consistency. Nevertheless, reaching orgasm every time is neither necessary nor necessarily better for a woman’s satisfaction, information that might help couples manage distress when orgasm does not occur and reduce a goal-oriented mindset that might hinder both orgasm and sexual pleasure.

## Introduction

Sexuality and sexual expression are an integral part of human experience, influencing physical, emotional, and relational well-being. The World Health Organization [WHO] has highlighted the importance of people’s ability to obtain a “satisfying and safe sex life” in “[enhancing] life and personal relations” (International Conference on Population and Development, 1994, as cited in World Health Organization & UNDP/UNFPA/UNICEF/WHO/World Bank Special Programme of Research, Development & Research Training in Human Reproduction, 2017, p. 2). Sexual intimacy plays an important role within romantic relationships, with many sex therapists viewing sexual fulfillment and relationship quality as interdependent factors (College of Sexual & Relationship Therapists, n.d.). An understanding of what constitutes good sex is key to the optimization of sexual functioning, as is determining whether this correlates for men and women.

Orgasms in particular have become synonymous with good sex, and it is no secret that women are less likely than men to orgasm in heterosexual relationships, a phenomenon widely termed the “orgasm gap” (Gesselman et al., 2024; Mahar et al., 2020; Wetzel & Sanchez, 2022). Research to date has indicated that this disparity may have implications for relationship and sexual satisfaction. In predominantly North American samples, a significant positive association has been found between sexual satisfaction and women’s orgasm likelihood, consistency, and frequency (Frederick et al., 2017; Haning et al., 2007; Leonhardt et al., 2018, 2023; Leavitt et al., 2021; Pascoal et al., 2018; Sprecher, 2002; Sprecher & Cate, 2004; Waterman & Chiauzzi, 1982) and between women’s orgasmic pleasure, consistency, and frequency with relationship satisfaction (Leonhardt et al., 2018; Leavitt et al., 2021; Mah & Binik, 2005). Sexual and relationship satisfaction, separate but related constructs in couples, have unsurprisingly been found to correlate, with a stronger correlation present for men (Byers, 2005; Christopher & Sprecher, 2000; Guo & Huang, 2005; Haavio-Mannila & Kontula, 1997; Purnine & Carey, 1997; Sprecher, 2002; Quinn-Nilas, 2019; Vowels & Mark, 2020). Longitudinal research suggests this relationship is dynamic and bidirectional, with sexual and relationship satisfaction influencing each other over time (Byers, 2005; Fallis et al., 2016; McNulty et al., 2016).

Gendered socio-sexual scripts profoundly shape expectations regarding sex and orgasm, often reinforcing traditional roles that impact sexual experiences and expectations (Simon & Gagnon, 1986; Wiederman, 2005). In Western contexts, these scripts frequently depict men as sexual initiators and women as passive participants, prioritizing vaginal-penile intercourse and male orgasm as markers of “real sex” (Jackson, 1984; Kiefer et al., 2006; McPhillips et al., 2001; Sanchez et al., 2012; Wiederman, 2005). Such narratives can diminish women’s sexual assertiveness, limiting orgasm likelihood and satisfaction, while portraying men as responsible for sexual outcomes (Kiefer et al., 2006; Lentz & Zaikman, 2021; Salisbury & Fisher, 2014). These traditional scripts often overlook the fact that women are more likely to achieve orgasm through a variety of sexual acts rather than vaginal-penile intercourse alone (Frederick et al., 2017; Herbenick et al., 2010). Media and pornography exacerbate these expectations, often depicting unrealistic sexual standards, with women’s orgasms overlooked or misrepresented, contributing to misunderstanding and relational conflicts (Cabrera & Ménard, 2013; Lavie-Ajayi & Joffe, 2009; Séguin et al., 2018). These gendered expectations highlight the pervasive cultural influences that shape sexual experiences, satisfaction, and interpretations.

The wider cultural context also plays a significant role in how people’s sexuality is expressed and their interpretation of the resulting outcomes (Bhutto et al., 2021; Zadeh et al., 2021), yet there is insufficient cross-cultural research to determine whether women’s orgasms universally correlate with women’s relationship satisfaction, or if this relationship is population specific. Aotearoa/New Zealand provides a unique context for exploring this dynamic, given the admixture of bicultural influences following colonization and recent addition of non-Western immigrant communities. Māori culture, rooted in the traditions of Aotearoa/New Zealand’s Indigenous people, historically embraced a diverse and open approach to sexuality (Binney, 1975; Bleys, 1995, as cited in Aspin & Hutchings, 2007; Broughton, 1996; Te Awekotoku, 1996, 2003, as cited in Aspin & Hutchings, 2007; Wallace, 2003). However, British colonization introduced conservative Victorian Christian values (Csako et al., 2022), creating a mix of liberal and restrictive attitudes toward sexual expression (Aspin & Hutchings, 2007). Similar changes from open to conservative attitudes toward sexuality occurred in other indigenous communities with the arrival of Europeans (Jacobs, 1997; Walters et al., 2006). Recent immigration has further enriched the cultural landscape, bringing diverse perspectives from global diasporas. Consequently, women’s sexual satisfaction within relationships in Aotearoa/New Zealand may reflect an interplay of traditional, colonial, and contemporary cultural influences, shaped by both individual and relational priorities. We might expect that the relationship between satisfaction and orgasm in such a context demonstrates significant variability, and that awareness of such variability may better inform therapeutic approaches taken toward sexuality in an era of increasingly multicultural contexts of practice.

Orgasms are not merely physiological events, but highly subjective experiences influenced by relational, emotional, and personality factors, in addition to sociocultural factors (Bentler & Peeler, 1979; Levin, 2004; Lousada & Angel, 2011; Mah & Binik, 2002; Zadeh et al., 2021). Variations in how orgasms are experienced and reported can be attributed to both inter- and intra-personal sexual responses, diverse motivations for sexual behavior, and varying expectations regarding women’s orgasm (Butler, 1976; Nicolson & Burr, 2003; Rowland & Gutierrez, 2017; Wetzel et al., 2022). The biopsychosocial context significantly influences both the experience and perception of orgasm. Unsurprisingly, the constructs and measurement of orgasm, sexual, and relationship satisfaction are nuanced and complex too (Hudson, 2013; Mah & Binik, 2002; Pascoal et al., 2014; Røysamb et al., 2014). As studies on the orgasm to date have generally focused on a single dimension and analyzed its relationship directly with sexual and/or relationship satisfaction in isolation of other orgasmic variables, much of the nuance of the orgasmic experience and impact of confounding variables may have been missed. By considering the multiple dimensions of the orgasmic experience and applying a stepwise logistic regression model, this research will try and establish which specific aspects of women’s orgasm best explain women’s sexual relationship satisfaction in an Aotearoa/New Zealand sample, and how these factors interact with each other. Further, data on age, partnered sex frequency, and self-rated importance of sex permit the exploration of whether these factors explain women’s relationship satisfaction better than women’s orgasmic pleasure alone.

## Materials and Methods

This study is part of a cross-cultural research project including the United States, Hungary, and Aotearoa/New Zealand. This study focuses on the data within the cultural context of Aotearoa/New Zealand, and data regarding the United States and Hungary have been presented elsewhere (e.g., Hevesi et al., 2024; Rowland et al., 2018).

### Participants

Participants were 483 New Zealand women who self-enrolled in the study. The inclusion criteria were gender and age, with the survey open to individuals aged 18 and older who identified as a woman, resided in Aotearoa/New Zealand, and answered relevant survey questions about their orgasm during partnered sex, relationship satisfaction and indicated they have had a sexual primary relationship (considered a relationship beyond sex) within the past 12 months, regardless of sexual orientation.

### Measures

A 42-item questionnaire developed by Rowland et al. (2018) to investigate which factors women consider important to their sexual health was utilized for data collection. The questionnaire has five principal sections covering sociodemographic and health information, the role of sex in the participants life, individual sexual response, orgasmic response during masturbation, and orgasmic response during partnered sex. This research project draws on answers to those question items concerning primary sexual relationship satisfaction, age, ethnocultural identification, educational level, menopause status, sexual orientation, gender identity, self-rated importance of sex and partnered sex frequency, orgasmic consistency, difficulty reaching orgasm, and self-rated orgasmic pleasure/satisfaction. Sociodemographic questions used either a fill-in-the-blank or multiple-choice format, with the option to elaborate where relevant. Frequency of sex response options were multiple choice. The remaining question items made use of a five- or ten-point Likert-type scale. Where relevant, questions specified responses pertaining to the past year and the current or most recent relationship/sexual partner. Tables 1 and 2 in the supplementary materials contains those question items selected for this study and the response options.

### Procedure

Ethical approval was obtained in New Zealand by AUTEC (application no. 18/96 “Good sex, bad sex: what factors are important for women”).

Online community-based convenience sampling of Aotearoa/New Zealand women was conducted in 2018 using Qualtrics survey software. Participants were informed that the study aimed to investigate women’s sexual health and problems to help clinicians develop better clinical interventions. A paid advertisement was promoted on social media platforms, including Facebook and Instagram, alongside flyers placed on university community notice boards. All participation was voluntary, anonymous, with no reimbursement offered for taking part. Prior to starting the survey, participants were informed of the purpose, written consent was gained, and eligibility was confirmed. During the survey, participants were made aware that they could opt out of answering certain questions and could withdraw at any point. Data were only stored following the final submission of the survey, and the privacy and confidentiality of participants’ information was protected by maintaining their anonymity.

### Data analysis

Descriptive statistics were used to describe sample sociodemographic characteristics and demonstrate the variance for questionnaire items While effort was made to retain meaningful categories, answers were collapsed into fewer categories where necessary to ensure sufficient numbers for between category analysis. In this process the outcome variable - primary sexual relationship satisfaction - was collapsed into the binary category of “satisfied” (Likert scale 4 and 5) and “not satisfied” (Likert scale 1, 2 and 3) to ensure that the model was sufficiently powered. A stepwise binary logistic regression model building exercise was thus deemed appropriate to elucidate the most fitting set of variables to explain primary sexual relationship satisfaction. Cross-tabulations were used to check adequate spread of the data between the outcome variable and explanatory variables or covariate answers. Where this was not the case, categories were further collapsed (see Table 2 in supplementary material for the recoding of variables and Tables 3, 4, and 5 for percentages of respondents in each category during cross-tabulation process).

Logistic regression analyses were first run with each predictor variable and covariate against the outcome variable. Those significantly associated with the outcome variables (*p* ≤ 0.2) were identified as potential candidates for the models. These selected predictor variables were tested for collinearity, and non-collinear covariates were then included in a stepwise logistic regression model that aimed to combine these variables and covariates in a holistic manner to best explain the outcome variables. This model proceeded by adding each predictor variable and covariate stepwise to the model, with a comparison being made to the model without the addition in order to determine whether each inclusion provided significantly more explanatory power. A significance level of *p* ≤ 0.05 was used for this latter process. Finally, the regression analysis was run in both directions to confirm the stability of the result.

Missing data were within an acceptable range: from 0.8% to 3.1% for sociodemographic covariates, and 0% to 1.0% for explanatory variables.

## Results

Sample socio-demographics are displayed in Table 1. Approximately half of the women were satisfied with their sexual relationship, with 56% of study participants selecting a response of 4 or 5 on the 5-point Likert scale.

**Table 1.** Sociodemographic Characteristics of Participants.

Sample characteristics	
Age in years, *M* (*SD*)	35.3 (11.6)
Age in years (*%*)	
18–24	20.3
25–34	30.8
35–44	25.9
45–54	17.3
55+	5.8
Menopause status (*%*)	
Pre-menopause	83.4
Perimenopause	7.2
Post-menopause	7.2
Does not apply	1.9
Ethnocultural identification (*%*)	
NZ Māori	7.6
NZ European/Pākehā	73.5
Pasifika	0.6
Asian	2.7
Latin American	0.2
European Other	14.9
Not clearly specified	0.6
Highest Educational Level (*%*)	
Primary school	0.2
Secondary school: high school/college	15.5
Technical college	2.9
Skill certification/other post high school technical degree	15.7
Some university	13.8
University degree: bachelor’s (undergraduate) degree	32.9
University degree: post-graduate degree, PhD, doctorate	18.9
Sexual orientation (*%*)	
Heterosexual	72.2
Homosexual	2.1
Bisexual	24.4
Demisexual	0.2
Pan-sexual	0.6
Asexual	0.2
Gender identity	
Cisgender woman	99.6
Transgender woman	0.2
Gender fluid	0.2

Table 2 shows the variables that met the threshold (*p* ≤ 0.2) to be considered candidates for the model. Inputting these candidates into the logistic regression model demonstrated that sexual relationship satisfaction was best explained by partnered sex orgasm consistency, frequency of sex, self-rated importance of sex, and age. This model was statistically significant (X2 (11, N = 351) = 140.19, *p* < 0.001), demonstrating that it could distinguish between women who were and were not satisfied in their sexual relationship. The model explained between 32.9% (Cox & Snell R^2^) and 47.8% (Nagelkerke R^2^) of the variance in sexual relationship satisfaction and correctly classified 84.3% of cases. Table 3 presents a full breakdown of the results of the combined logistic regression model.

**Table 2.** Individual Associations of Predictor Variables and Covariates with Sexual Relationship Satisfaction Among Aotearoa/New Zealand Women.

	Satisfied in relationship	OR (95% CI)	*p* value
	%		
Sociodemographic factor			
Age (years)			
18–24	91	1.00	–
25–44	70	0.25 (0.10–0.60)	–
45+	65	0.20 (0.08–0.51)	**0.003** * *
Non-orgasmic specific sex factors			
Self-rated importance of sex			
Low	49	1.00	–
Moderate	73	2.82 (1.42–5.58)	–
High	79	4.00 (2.13–7.50)	**<0.001** ** *
Frequency of sex			
2+ times per week	85	1.00	–
About weekly	84	0.88 (0.41–1.87)	–
1–2 per month	66	0.33 (0.18–0.63)	–
<1 times per month	17	0.04 (0.01–0.09)	**<0.001** ** *
Partnered sex orgasm factors			
Orgasm consistency			
Never-almost never	41	1.00	–
Infrequent	59	2.08 (0.99–4.40)	–
Around half the time	82	6.60 (2.70–16.16)	–
Often	88	10.71 (4.78–24.01)	–
Almost always-always	85	8.54 (4.27–17.08)	**<0.001** ** *
Orgasmic difficulty			
Almost never	84	1.00	–
Rarely	78	0.69 (0.31–1.54)	–
Sometimes	84	1.04 (0.46–2.32)	–
Often-almost always	60	0.28 (0.14–0.55)	–
Never orgasm	35	0.11 (0.04–0.26)	**<0.001** ** *
Orgasmic pleasure			
Never orgasm	52	1.00	
Low-moderate	60	1.39 (0.67–2.90)	–
High	77	3.15 (1.51–6.58)	–
Very high	83	4.67 (2.35–9.28)	**<0.001** ** *

**p < 0.01. ***p < 0.001.

OR: odds ratio; CI: confidence interval.

**Table 3.** Logistic Regression Model Showing the Variables that Best Predicted Sexual Relationship Satisfaction.

	Multiple variable relationship model	
	OR (95% CI)	*p* value
Self-rated importance of sex		
Low	1.00	–
Moderate	3.34 (1.42–7.88)	–
High	3.31 (1.52–7.21)	**0.006** * *
Frequency of sex		
2+ times per week	1.00	–
About weekly	1.02 (0.43–2.45)	–
1–2 per month	0.35 (0.17–0.74)	–
<1 times per month	0.04 (0.02–0.13)	**<0.001** ** *
Orgasm consistency		
Never-almost never	1.00	–
Infrequent	2.32 (0.92–5.87)	–
Around half the time	7.96 (2.72–23.34)	–
Often	11.77 (4.41–31.42)	–
Almost always-always	10.30 (4.42–24.00)	**<0.001** ** *
Age (years)		
18–24	1.00	–
25–44	0.30 (0.11–0.84)	–
45+	0.26 (0.08–0.80)	**0.048** *

*p < 0.05. **p < 0.01. ***p < 0.001.

OR: odds ratio; CI: confidence interval.

Likelihood of sexual relationship satisfaction increased with orgasm consistency up to a level where women reported sexual activity often ended in orgasm; the latter were almost 12 times more likely to be satisfied compared to women who recorded sexual activity never-to-almost never ending in orgasm (OR = 11.77, 95%CI [4.41, 31.42]). A reduction in the likelihood of relationship satisfaction was observed after this point, with women who answered sexual activity almost-always to always ending in orgasm just over ten times more likely to be satisfied (OR = 10.30, 95%CI [4.42, 24.00]). Less frequent partnered sex was associated with a lower likelihood of sexual relationship satisfaction, and women who reported having partnered sex one to two times a month were approximately three times less likely to be satisfied than women who reported having sex two or more times a week (OR = 0.35, 95%CI [0.17, 0.74]). This effect was even more pronounced for women who reported having sex less than once a month, who were 25 times less likely to be satisfied compared to women who reported having sex two or more times a week (OR = 0.04, 95%CI [0.02, 0.13]). The likelihood of sexual relationship satisfaction decreased with age, with women aged 25 to 44 more than three times less likely to be satisfied (OR = 0.30, 95%CI [0.11, 0.84]) and women aged over 45 almost four times less likely to be satisfied (OR = 0.26, 95%CI [0.08, 0.80]) compared to women aged 18 to 24. Finally, women were over three times more likely to be satisfied in their sexual relationship if they reported sex to be of moderate importance (OR = 3.34, 95%CI [1.42, 7.88]) and high importance (OR = 1.52, 95%CI [0.02, 7.21]) when compared with women who reported sex to be of low importance.

## Discussion

Consistent with studies conducted outside of Aotearoa/New Zealand (e.g., Leavitt et al., 2021; Leonhardt et al., 2018; Mah & Binik, 2005), more orgasms and more orgasmic pleasure related to more sexual relationship satisfaction, but only up to a point. Orgasm consistency, the strongest orgasmic predictor of satisfaction, peaks for those reporting frequent orgasm. This aligns with the curvilinear relationship described by Leavitt et al. (2021) where satisfaction plateaued at 61 to 80% orgasm consistency. Leavitt et al. (2021) hypothesized that high orgasm consistency might be best attributed to individual sexual response rather than partner or relational factors, and therefore that they attribute their pleasure accordingly. An alternative explanation is that women may be more likely to stay in a relationship should it provide very regular orgasmic pleasure even if it is otherwise lacking, although there is no research yet to corroborate this conjecture.

Non-orgasmic factors, namely frequency of sex, self-rated importance of sex, and age, also significantly relate to satisfaction, particularly the frequency of partnered sex; women having sex less frequently than once a week were far less likely to be satisfied in their sexual relationships. This is consistent with prior research demonstrating a relationship between sexual satisfaction, a correlate of relationship satisfaction, and frequency of sex (e.g., Frederick et al., 2017; McNulty et al., 2016; Roels & Janssen, 2020; Smith et al., 2011). The directionality of this relationship cannot be determined in this study; however, longitudinal research has indicated it to be dynamic and bidirectionally positive (McNulty et al., 2016; Quinn-Nilas, 2020).

Interestingly, women who rated sex as of low importance were less likely to be satisfied. Although again we cannot establish directionality in this study, it seems unlikely that low self-rated importance of sex causes low sexual satisfaction; were sex to be unimportant for women, then it would follow that they would be more easily satisfied in their sexual relationship, or alternatively no association would exist. Therefore, the presence of an association here suggests that low satisfaction in their sexual relationship may cause women to self-rate the importance of sex as low as a coping strategy. This finding aligns with cognitive dissonance theory (Festinger, 1962), whereby if the behavior (in this case, remaining in an unsatisfying sexual relationship) conflicts with the person’s values (such as valuing sex as important), the individual will change to minimize the associated negative affect (as illustrated in Festinger & Carlsmith, 1959). This implies that a women’s low self-rated importance of sex may not always be an accurate measure and may instead reflect their level of sexual relationship satisfaction.

The presence of the non-orgasmic sexual variables in the final model suggests that sex may provide benefits beyond simply orgasmic pleasure; if only orgasms were important, then these variables would not provide significantly greater explanatory power to the model. This fits with research demonstrating that both men and women do not necessarily view orgasm as a requisite element for their own sexual pleasure – indeed, many view affection, sensuality, and intimacy as of equal or greater importance (Bolsø, 2005; Potts et al., 2006; Lavie & Willig, 2005; Nicolson & Burr, 2003), with closeness and dyadic mutuality rated as the most important elements of partnered sex (Rowland et al., 2019). The data conflict with the common Western socio-sexual script - the orgasm imperative - that places orgasm as the goal of sex (Frith, 2015). The orgasm imperative has been critiqued for limiting the perception of sexual satisfaction and portraying the failure to reach orgasm as a problem, which can result in pressure to achieve orgasm and distress and relationship difficulty when it does not occur (Cacchioni, 2015; Denman, 2004; Lavie-Ajayi, 2005; Morgentaler, 2013; Tiefer, 2004). Therefore, while orgasms are associated with women’s sexual relationship satisfaction, sex beyond orgasmic pleasure is also significantly associated with women’s sexual relationship satisfaction.

Women aged 18 to 24 were most satisfied with sexual aspects of their relationships. This could be related to the age-related decline in biological sexual function (Hayes & Dennerstein, 2005). However, if there was a purely biological cause one would expect the greatest decrease to occur between 25 to 44 years and 45 plus years age groups, since the latter group includes post-menopausal women, who are the most likely to have experienced the greatest hormonal changes related to sexual functioning (Kingsberg, 2002). The impact of menopause, however, may be somewhat countered by the fact that women’s sexual satisfaction can improve with aging due to psychological factors, with increases observed in sexual assertiveness, comfort with sex, and sexual satisfaction (Miller, 2019). In addition, the concept of spontaneous sexual drive as the main motivation for sexual behavior may not be accurate for women (Basson, 2001); many women are more strongly motivated by a desire to increase emotional connection or dyadic mutuality (Mayland, 2005), and thus a biological decrease in sexual desire may be of little consequence, at least in some women. Therefore, the results here might be better explained by sexuality development; young women on average may have lower orgasmic expectations as, for many, the period between 18 and 24 years can be a time of emerging sexuality (Salisbury & Fisher, 2014). Fitting with this, lower orgasm consistency and higher orgasmic latency has been found among younger adults compared with their older counterparts (Boroditsky et al., 1999; Hevesi et al., 2024; Laumann et al., 1994). As such, women at this age may be more inclined to view infrequent orgasms as a normal part of their sexual learning (O’Sullivan & Majerovich, 2008) and may therefore be more satisfied due to their lower sexual expectations.

While all orgasm variables were related to satisfaction, orgasm consistency was the only variable included in the final model. It is unclear why this orgasm variable was the most important for sexual relationship satisfaction. A possible explanation for its significance over difficulty reaching orgasm lies in the differences in how the two question items were framed. These questions are very similar in what they ask and are nearly a reverse-scoring of each other. However, difficulty reaching orgasm implies that the individual is trying to reach orgasm; if a woman is not actively trying to reach orgasm during sexual activity, then no difficulty is experienced whether or not she orgasms. Therefore, while difficulty reaching orgasm may be measuring the consistency of orgasms for some women, namely those who aim to reach orgasm every time, for other women it could be a measure of how often women aim to reach orgasm. This fits with the research showing that reaching orgasm is not always considered necessary for sexual enjoyment (Bolsø, 2005; Potts et al., 2006; Lavie & Willig, 2005; Nicolson & Burr, 2003). An explanation for why orgasm consistency is more significant than orgasmic pleasure rating might be that the latter may be more dependent on individual sexual response and the individual’s ability to remain present in the moment (Leavitt et al., 2021; Mayland, 2005). Therefore, orgasmic pleasure may be more likely to be attributed to the self and consistency to the relationship and partner factors (Bhutto et al., 2021).

This study indicates that women in Aotearoa/New Zealand experience a similar pattern between orgasmic pleasure and relationship satisfaction when compared to women in North America. Given that the demographics of our sample resemble those of studies performed in North America (with approximately three quarters of respondents identifying as NZ Pākehā/Caucasian), this is to be expected. While it unfortunately prevents an assessment of the impact of diversity on orgasmic pleasure and relationship satisfaction, it does give us reason to believe that the other findings of this study may be applicable to Western populations outside of Aotearoa/New Zealand. Our study would have required a larger sample to assess for differences among various demographic groups, and collecting such data offers an avenue for future exploration.

## Strengths and limitations

This study builds upon prior research by Csako et al. (2022), which focused on women’s masturbation practices, to begin forming a comprehensive picture of Aotearoa/New Zealand women’s sexuality in both partnered and solitary sexual contexts. The findings offer an initial understanding of how orgasmic pleasure relates to relationship satisfaction among Aotearoa/New Zealand women. However, there are significant limitations: the directionality of the relationship cannot be inferred, cross-cultural applicability of the original survey designed and validated within Hungary to the Aotearoa/New Zealand context is unknown, the generalizability of the sample is questionable with the low representation of minority groups, and non-probability sampling and sexuality research lends itself to selection bias (Catania et al., 1990; Sharma, 2017; Wiederman, 1999). In addition, the single measure question items used in this study are susceptible to self-report bias (Brenner & DeLamater, 2016). Staying in a relationship that an individual openly admits is unsatisfying would likely create cognitive dissonance (Festinger, 1962). Therefore, women in these situations might be more inclined to report higher levels of satisfaction to create cognitive consonance (Festinger, 1962). The use of validated psychometric assessments on sexual satisfaction and relationship satisfaction, such as the Index of Sexual Satisfaction (Hudson, 2013) and Relationship Satisfaction scale [RS] (Røysamb et al., 2014), may reduce this effect in future research; such measures use questions related to the construct of satisfaction without explicitly enquiring about it. Furthermore, quantitative research alone on the complex topic of human sexuality and relationships is of limited use for in depth analysis.

## Conclusion

Women’s orgasms are connected to their sexual relationship satisfaction in Aotearoa/New Zealand. As this study aligns with patterns observed in Western samples abroad, the results are likely applicable to Western populations outside Aotearoa/New Zealand. Therefore, if a sex and relationship therapist is working with a Western woman seeking to improve her sexual relationship satisfaction, the findings of this study would support focusing the intervention on having more regular sex and/or enhancing orgasm consistency rather than strategies to increase the pleasure derived from their orgasms. We cannot infer the direction the relationship, but based on similar research (see Frederick et al., 2017), the relationship may be dynamic and bidirectional, where orgasm improves relationship satisfaction and relationship satisfaction in turn improves orgasm consistency. Perhaps the key finding here is that orgasms, while important, are not the only significant element of sex for women’s sexual relationship satisfaction and that reaching orgasm every time is not required for, or necessarily more positively associated with, a woman’s satisfaction. This information conveyed to individuals, or couples may help reduce the distress when orgasm does not occur and remove the performance and goal orientation of sex that can be a hindrance to both orgasms and sexual pleasure (Cacchioni, 2015; Denman, 2004; Lavie-Ajayi, 2005; Morgentaler, 2013; Tiefer, 2004).

## Supplementary Material

Supplementary Materials.docx

## Data Availability

Raw data were generated at Auckland University of Technology. Derived data supporting the findings of this study are available from the corresponding author A.J. on request.
