# Supplementary material for: The Satisfaction of Women’s Orgasms: The Relationship Between Women’s Orgasmic Pleasure and Sexual Relationship Satisfaction in Aotearoa/New Zealand
Source: Int J Sex Health. 2025 Feb 22;37(2):251–62. doi: 10.1080/19317611.2025.2464535 (PMC12091931; doi:10.1080/19317611.2025.2464535)
Supplement: Supplementary Materials.docx [file WIJS_A_2464535_SM6991.docx]

Supplementary Materials

**Table 1**
*Survey Items and Corresponding Codes*

| Question Item | Code |
| --- | --- |
| Outcome variable 1: How satisfied are you with your primary *sexual* relationship, that is, with the relationship you consider to be most significant to you? | Sexual relationship satisfaction |
| Age | Age |
| Please rate the importance of sex in your life? | Importance of sex |
| Considering your sexual history with your current or most recent ongoing partner, how often do you or did you have sex with your partner? | Frequency of sex |
| Estimate how often sexual activity with your partner ends in (or ended in) orgasm by placing an X on the line below. Base your answer on your current or most recent sexual partner. If you have not had a sexual partner or have not had any sexual activities with a partner, select N/A (not applicable). | Orgasm consistency |
| *When you have (or have had) sex with a partner*, how pleasurable or satisfying would you rate your typical orgasm. | Orgasmic pleasure |
| *When you have (or have had) sex with your partner*, do (or did) you have problems reaching orgasm? | Orgasmic difficulty |

**Table 2**
*Collapse and Recoding of Question Variables for Logistic Regression Analysis*

| Question item | Original response options | Recoded responses (contrast code) |
| --- | --- | --- |
| Sexual relationship satisfaction | 1 = not at all satisfied to 5 = very satisfied | 1 to 3 = not satisfied (0); 4 to 5 = satisfied (1) |
| Age | Continuous | 18-24 years (1); 25-44 years (2); 45 plus years (3) |
| Importance of sex | 1 = not at all important to 5 = very important | 1 to 2 = low (1); 3 = moderate (2); 4 = high (3) |
| Frequency of sex | I do not have or have not had a sexual partner; one or more times daily; 2-3 times per week; about 1 time per week; about 1 time every 2 weeks; about once a month; less than once a month; almost never; never | I do not have or have not had a sexual partner = N/A (N/A); one or more times daily and 2-3 times per week = 2+ p/week (1); about 1 time per week = about weekly (2); about 1 time every 2 weeks and about once a month = 1-2 times p/month (3); less than once a month, almost never, and never = <1 p/month (4) |
| Orgasm consistency | 1 = never to 10 = always; N/A | 1 to 2 = never – almost never (1); 3 to 4 = infrequent (2); 5 to 6 = about half the time (3); 7 to 8 = often (4); 9 to 10 =almost always – always (5) |
| Orgasmic difficulty | I do not reach orgasm with my partner; 1 = almost never to 5 = almost always | 1 = almost never (1); 2 = rarely (2); 3 = sometimes (3); 4 to 5 = often-always (4); I do not reach orgasm with my partner = never orgasm (5) |
| Orgasmic pleasure | 1 = not satisfying to 5 = very satisfying; I do not reach orgasm with my partner | I do not reach orgasm with my partner = never orgasm (N/A); 1 to 3 = low-moderate satisfaction (1); 4 = high satisfaction (2); 5 = very high satisfaction (3) |

**Table 3**
*Cross-Tabulation of Predictor Variable Percentages with Sexual Relationship Satisfaction Among Aotearoa/New Zealand Women, using Original Survey Responses for both Outcome and Predictor Variables*

|  | Sexual relationship satisfaction (%) 1=not at all satisfied 5=very satisfied | | | | |  |
| --- | --- | --- | --- | --- | --- | --- |
|  | 1 | 2 | 3 | 4 | 5 |  |
| *Sociodemographic factor* |  |  |  |  |  |  |
| Age (years) |  |  |  |  |  |  |
| 18-24 | 0.4 | 0.9 | 4.5 | 7.9 | 6.6 |  |
| 25-34 | 2.1 | 4.5 | 8.1 | 9.0 | 7.1 |  |
| 35-44 | 3.0 | 3.4 | 6.0 | 7.9 | 5.6 |  |
| 45-54 | 1.9 | 2.6 | 3.8 | 4.9 | 4.1 |  |
| 55+ | 0.9 | 0.6 | 1.3 | 1.3 | 1.7 |  |
| *Non-orgasmic specific sex factors* |  |  |  |  |  |  |
| Self-rated importance of sex |  |  |  |  |  |  |
| 1 = not at all important | 1.0 | 1.7 | 0.8 | 0.4 | 0.4 |  |
| 2 | 1.0 | 2.1 | 2.7 | 1.7 | 0.6 |  |
| 3 | 2.7 | 3.1 | 7.3 | 8.5 | 3.7 |  |
| 4 | 2.1 | 2.9 | 6.2 | 11.2 | 9.3 |  |
| 5 = very important | 1.5 | 2.3 | 6.6 | 9.5 | 10.8 |  |
| Frequency of sex |  |  |  |  |  |  |
| One or more times daily | 0 | 0.6 | 0.6 | 3.7 | 5.0 |  |
| 2-3 times per week | 1.0 | 3.3 | 7.5 | 17.4 | 12.6 |  |
| About 1 time per week | 1.2 | 1.2 | 6.2 | 6.6 | 4.8 |  |
| About 1 time every 2 weeks | 0.8 | 2.9 | 3.9 | 2.3 | 1.8 |  |
| About once a month | 0.8 | 1.2 | 4.1 | 1.0 | 0.4 |  |
| Less than once a month | 1.7 | 2.5 | 1.0 | 0.2 | 0 |  |
| Almost never | 2.5 | 0.2 | 0.2 | 0 | 0.2 |  |
| Never | 0.2 | 0 | 0 | 0 | 0 |  |
| *Partnered sex orgasm factors* |  |  |  |  |  |  |
| Orgasm consistency |  |  |  |  |  |  |
| 1 = never | 2.9 | 1.9 | 1.2 | 1.9 | 1.0 |  |
| 2 | 1.5 | 2.9 | 1.9 | 1.2 | 0.4 |  |
| 3 | 0.8 | 1.0 | 2.9 | 1.0 | 0.6 |  |
| 4 | 0.4 | 1.7 | 0.6 | 1.0 | 0.2 |  |
| 5 | 0.2 | 0.4 | 1.9 | 2.1 | 1.2 |  |
| 6 | 0 | 1.0 | 2.1 | 1.5 | 0.2 |  |
| 7 | 0.4 | 0.4 | 2.9 | 3.7 | 1.7 |  |
| 8 | 0.8 | 0..41 | 2.7 | 5.8 | 3.7 |  |
| 9 | 0.6 | 0.8 | 3.5 | 5.8 | 7.9 |  |
| 10 = always | 0.6 | 1.5 | 3.9 | 7.3 | 7.9 |  |
| Orgasmic difficulty |  |  |  |  |  |  |
| 1 = almost never | 1.0 | 2.5 | 6.2 | 12.2 | 14.5 |  |
| 2 | 1.2 | 1.5 | 3.9 | 5.8 | 4.6 |  |
| 3 | 0.4 | 2.1 | 6.2 | 7.3 | 2.3 |  |
| 4 | 1.2 | 2.3 | 2.5 | 2.7 | 1.5 |  |
| 5 = almost always | 1.5 | 2.5 | 3.1 | 2.7 | 1.5 |  |
| I do not reach orgasm with my partner | 2.9 | 1.2 | 1.7 | 0.6 | 0.6 |  |
| Orgasmic pleasure |  |  |  |  |  |  |
| I do not reach orgasm with my partner | 2.9 | 2.3 | 3.3 | 2.3 | 0.8 |  |
| 1 = not satisfying | 0.4 | 0 | 0.2 | 0 | 0.2 |  |
| 2 | 0.4 | 0.4 | 1.2 | 0.2 | 0.2 |  |
| 3 | 0.8 | 3.3 | 3.3 | 3.1 | 0.6 |  |
| 4 | 1.0 | 3.1 | 6.0 | 8.1 | 3.3 |  |
| 5 = very satisfying | 2.5 | 2.9 | 9.5 | 17.6 | 19.5 |  |

*Note*. Age, originally measured as a continuous variable, has been categorised for presentation in this table.

**Table 4**
*Cross-tabulation of Predictor Variables Original Survey Responses with Dichotomised Sexual Relationship Satisfaction Among Aotearoa/New Zealand Women*

|  | Satisfied | |  | Not Satisfied | |  |
| --- | --- | --- | --- | --- | --- | --- |
|  | N | % |  | N | % |  |
| *Sociodemographic factor* |  |  |  |  |  |  |
| Age (years) |  |  |  |  |  |  |
| 18-24 | 68 | 14.5 |  | 27 | 5.8 |  |
| 25-34 | 75 | 16.0 |  | 69 | 14.7 |  |
| 35-44 | 63 | 13.5 |  | 58 | 12.4 |  |
| 45-54 | 42 | 9.0 |  | 39 | 8.3 |  |
| 55+ | 14 | 3.0 |  | 13 | 2.8 |  |
| *Non-orgasmic specific sex factors* |  |  |  |  |  |  |
| Self-rated importance of sex |  |  |  |  |  |  |
| 1 = not at all important | 4 | 0.8 |  | 17 | 3.5 |  |
| 2 | 11 | 2.3 |  | 28 | 5.8 |  |
| 3 | 59 | 12.2 |  | 63 | 13.0 |  |
| 4 | 99 | 20.5 |  | 54 | 11.2 |  |
| 5 = very important | 98 | 20.3 |  | 50 | 10.4 |  |
| Frequency of sex |  |  |  |  |  |  |
| One or more times daily | 42 | 8.7 |  | 6 | 1.2 |  |
| 2-3 times per week | 245 | 30.0 |  | 57 | 11.8 |  |
| About 1 time per week | 55 | 11.4 |  | 42 | 8.7 |  |
| About 1 time every 2 weeks | 20 | 4.1 |  | 37 | 7.7 |  |
| About once a month | 7 | 1.4 |  | 30 | 6.2 |  |
| Less than once a month | 1 | 0.2 |  | 25 | 5.2 |  |
| Almost never | 1 | 0.2 |  | 14 | 2.9 |  |
| Never | 0 | 0.0 |  | 1 | 0.2 |  |
| *Partnered sex orgasm factors* |  |  |  |  |  |  |
| Orgasm consistency |  |  |  |  |  |  |
| 1 = never | 14 | 2.9 |  | 29 | 6.0 |  |
| 2 | 8 | 1.7 |  | 30 | 6.2 |  |
| 3 | 8 | 1.7 |  | 23 | 4.8 |  |
| 4 | 6 | 1.2 |  | 13 | 2.7 |  |
| 5 | 16 | 3.3 |  | 12 | 2.5 |  |
| 6 | 8 | 1.7 |  | 15 | 3.1 |  |
| 7 | 26 | 5.4 |  | 18 | 3.7 |  |
| 8 | 46 | 9.5 |  | 19 | 3.9 |  |
| 9 | 66 | 13.7 |  | 24 | 5.0 |  |
| 10 = always | 73 | 15.1 |  | 29 | 6.0 |  |
| Orgasmic difficulty |  |  |  |  |  |  |
| 1 = almost never | 129 | 26.7 |  | 47 | 9.7 |  |
| 2 | 50 | 10.4 |  | 32 | 6.6 |  |
| 3 | 46 | 9.5 |  | 42 | 8.7 |  |
| 4 | 20 | 4.1 |  | 29 | 6.0 |  |
| 5 = almost always | 20 | 4.1 |  | 34 | 7.0 |  |
| I do not reach orgasm with my partner | 6 | 1.2 |  | 28 | 5.8 |  |
| Orgasmic pleasure |  |  |  |  |  |  |
| I do not reach orgasm with my partner | 15 | 3.1 |  | 41 | 8.5 |  |
| 1 = not satisfying | 1 | 0.2 |  | 3 | 0.6 |  |
| 2 | 2 | 0.4 |  | 10 | 2.1 |  |
| 3 | 18 | 3.7 |  | 36 | 7.5 |  |
| 4 | 55 | 11.4 |  | 49 | 10.1 |  |
| 5 = very satisfying | 179 | 37.1 |  | 72 | 14.9 |  |

**Table 5**
*Cross-Tabulation of Predictor Variable and Covariates with Dichotomised Sexual Relationship Satisfaction Among Aotearoa/New Zealand Women, using the Collapsed and Recoded Variables*

|  | Satisfied | |  | Not Satisfied | |  |
| --- | --- | --- | --- | --- | --- | --- |
|  | N | % |  | N | % |  |
| *Sociodemographic factor* |  |  |  |  |  |  |
| Age (years) |  |  |  |  |  |  |
| 18-24 | 68 | 14.5 |  | 27 | 5.8 |  |
| 25-44 | 138 | 29.5 |  | 127 | 27.1 |  |
| 45+ | 56 | 12.0 |  | 52 | 11.1 |  |
| *Non-orgasmic specific sex factors* |  |  |  |  |  |  |
| Self-rated importance of sex |  |  |  |  |  |  |
| Low | 15 | 3.1 |  | 45 | 9.3 |  |
| Moderate | 59 | 12.2 |  | 63 | 13.0 |  |
| High | 197 | 40.8 |  | 104 | 21.5 |  |
| Frequency of sex |  |  |  |  |  |  |
| 2+ times per week | 187 | 38.7 |  | 63 | 13.0 |  |
| About weekly | 55 | 11.4 |  | 42 | 8.7 |  |
| 1-2 per month | 27 | 5.6 |  | 67 | 13.9 |  |
| <1 times per month | 2 | 0.4 |  | 40 | 8.3 |  |
| *Partnered sex orgasm factors* |  |  |  |  |  |  |
| Orgasm consistency |  |  |  |  |  |  |
| Never-almost never | 22 | 4.6 |  | 59 | 12.2 |  |
| Infrequent | 14 | 2.9 |  | 36 | 7.5 |  |
| Around half the time | 24 | 5.0 |  | 27 | 5.6 |  |
| Often | 72 | 14.9 |  | 37 | 7.7 |  |
| Almost always-always | 139 | 28.8 |  | 53 | 11.0 |  |
| Orgasmic difficulty |  |  |  |  |  |  |
| Almost never | 129 | 26.7 |  | 47 | 9.7 |  |
| Rarely | 50 | 10.4 |  | 32 | 6.6 |  |
| Sometimes | 46 | 9.5 |  | 42 | 8.7 |  |
| Often-almost always | 40 | 8.3 |  | 63 | 13.0 |  |
| Never orgasm | 6 | 1.2 |  | 28 | 5.8 |  |
| Orgasmic pleasure |  |  |  |  |  |  |
| Never orgasm | 15 | 3.1 |  | 41 | 8.5 |  |
| Low-moderate | 3 | 0.6 |  | 13 | 2.7 |  |
| High | 18 | 3.7 |  | 36 | 7.5 |  |
| Very high | 234 | 48.6 |  | 121 | 25.2 |  |
